# Minimal Blood Pressure Effects of Intranasal Etripamil for Paroxysmal Supraventricular Tachycardia

**DOI:** 10.1016/j.jacadv.2026.102868

**Published:** 2026-06-10

**Authors:** James E. Ip, Peter Noseworthy, Farhad Rafii, Jonathan P. Piccini, Narendra Singh, Bruce Stambler, Silvia Shardonofsky, David B. Bharucha, A. John Camm

**Affiliations:** a Weill Cornell Medicine, New York Presbyterian Hospital, New York, New York, USA; b Mayo Clinic, Rochester, Minnesota, USA; c Interventional Cardiology Medical Group, West Hills, California, USA; d Duke University, Durham, North Carolina, USA; e NSC Research Center, Johns Creek, Georgia, USA; f Piedmont Heart Institute, Atlanta, Georgia, USA; g Milestone Pharmaceuticals, Charlotte, North Carolina, USA; h City St George's University of London, London, United Kingdom

**Keywords:** blood pressure, calcium channel blocker, etripamil, nasal spray, paroxysmal supraventricular tachycardia, self-administered

Etripamil (CARDAMYST), a novel intranasal nondihydropyridine L-type calcium channel blocker (CCB), is U.S. Food and Drug Administration-approved as the first and only self-administered treatment for adults with symptomatic paroxysmal supraventricular tachycardia (PSVT).^1^ A phase 3 trial of etripamil for atrial fibrillation with rapid ventricular rate is planned to start in 2026 (NCT06716021). Given the unique, intranasal self-administration of etripamil, the propensity of intravenous (IV) and oral (eg, pill-in-pocket) CCBs to cause adverse events of hypotension and syncope, and the poor efficacy of oral treatments, this analysis aimed to further explore the potential impact of etripamil on blood pressure (BP) and related cardiovascular safety.

**What is the clinical question being addressed?**

Is intranasal calcium channel blocker etripamil (CARDAMYST) for paroxysmal supraventricular tachycardia associated with hypotension or syncope?

**What is the main finding?**

Etripamil exhibits minimal blood pressure effects; hypotension and syncope occurred rarely, 0.4% and 0.2%, respectively, among >1,600 unique clinical trial participants.

The phase 3 etripamil PSVT trials included NODE-301 parts 1 to 3 (NCT03464019), NODE-302 (NCT03635996), and NODE-303 (NCT04072835). These studies, conducted from 2018 to 2023 in the United States, Canada, Europe, and South America, included double-blind randomized controlled and open-label designs. The pivotal RAPID (NODE-301 Part 2) study demonstrated that etripamil achieved significantly higher conversion rates than placebo (64% vs 31%; HR: 2.62; *P* < 0.0001) by 30 minutes following administration and a markedly shorter median time-to-conversion (17.2 vs 53.5 minutes).^2^ The group of clinical studies showed consistent efficacy, safety, and tolerability of etripamil for acute PSVT treatment.^1^ This is the first report to assess specific BP effects of etripamil, and to provide case-level analysis of hypotension- and syncope-related treatment emergent adverse events (TEAEs). This analysis complied with the Declaration of Helsinki, the study protocols were approved by the ethics committee of each site, and all patients provided informed written consent. Specific related information is previously published.^2^^,^^3^

A total of 1,107 patients received a test dose (TD) of etripamil whereas in sinus rhythm, with either a single- (70 mg by NODE-301 part 1 patients) or repeat-dose (70 mg ×2, 10 minutes apart by NODE-301 parts 2-3 patients). BP and heart rate measurements were obtained every 5 minutes post-TD for 30 minutes (after a single dose) or 45 minutes (after a repeat-dose). TD failure, defined as intolerance prompting study discontinuation before randomization, occurred in 16 patients (1.4%), primarily due to nasal symptoms (mild and transient).

Minimal change from baseline BP was observed after TD of etripamil in sinus rhythm (Figure 1). Mean systolic blood pressure (SBP) change was 1.8 mm Hg (SD: 11.2) over 30 minutes for single 70 mg dose (N = 438-440) (Figure 1A) and 0.0 mm Hg (SD: 12.0) over 45 minutes for repeat doses (N = 709-714) (Figure 1B); patient numbers varied slightly across time points due to recorded data availability. Within-group paired analyses showed small but statistically significant increases in diastolic BP at most time points (*P* < 0.001) and sporadic, inconsistent changes in SBP, with the magnitude and pattern of effects not indicating clinically meaningful hemodynamic impact. Heart rate monitored during TD while in sinus rhythm showed minimal mean change from baseline, consistent with the effects observed for BP.

**Figure 1:**
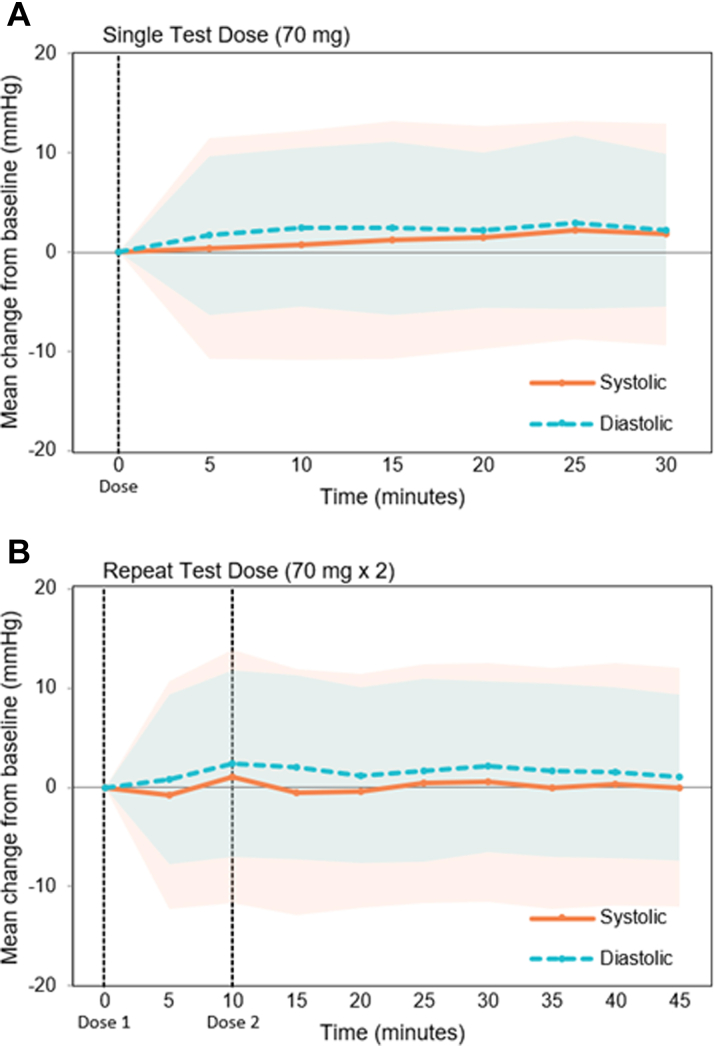
Change in Blood Pressure Following Etripamil Nasal Spray Test Dose The mean change from baseline in systolic (orange, solid) and diastolic (blue, dashed) blood pressure (BP) is shown with shaded bands indicating respective SD. (A) Single (70 mg, N = 438-440) and (B) repeat dosing (70 mg ×2, N = 709-714; second dose at 10 minutes) were evaluated in patients in sinus rhythm from NODE-301 parts 1 to 3. Vertical dashed lines indicate dosing time.

Among the 1,610 patients across the NODE-301, -302, and -303 phase 3 trials, 7 hypotension events (0.4%) were reported including 5 TD failures, with SBP reduction to <80 mm Hg or by 40 mm Hg if a baseline of >100 mm Hg or SBP reduction to <75 mm Hg if a baseline of 90 to 100 mm Hg; 3 of 5 cases (0.2%) had associated symptoms; 40% female; mean age 54 years (range: 35-71 years); nadir BP range, systolic, 53 to 88 mm Hg, and diastolic, 28 to 65 mm Hg. Two events occurred during SVT: 1) patient presented to an emergency department with SVT (∼187 beats per minute [bpm]) >1 hour after 1 dose of etripamil (treated with electrical cardioversion); and 2) another patient presented to an emergency department with SVT at 185 bpm ∼6 hours after 1 dose of etripamil (treated with IV adenosine). Due to the prolonged times between drug administration and observed hypotension during persistent SVT, both cases were judged as unlikely to be directly caused by etripamil.

We also report an analysis of TEAEs within 24 hours of etripamil administration of hypotension or syncope among the 1,610 unique patients across all phase 3 trials. The 7 TEAEs of hypotension have been described previously. Syncope was reported in 4 (0.2%) patients, with 2 (0.1%) events occurring before etripamil administration and thus were unrelated to the drug. One patient in NODE-303 reported “syncope” approximately 5 minutes after administering a single etripamil dose during SVT at ∼180 bpm, which did not terminate; however, the medical record showed no evidence or report of loss of consciousness. A second TEAE of syncope was reported in a patient in RAPID Extension (NODE-301, part 3) who had PSVT at ∼220 bpm, administered 2 doses of etripamil, after which there was brief loss of consciousness. On regaining consciousness, SVT was ongoing and terminated 11 minutes uneventfully after the second etripamil dose. This event was classified as mild and possibly related to etripamil.

In a prior report of NODE-303, the definition of TEAE was expanded, for conservatism, to include any patient-reported adverse events that may have occurred as early as 12 hours before drug administration.^3^ Under this broadened criterion, the 2 episodes of hypotension that occurred >1 and 6 hours after etripamil, and 2 reports of syncope that occurred *before* drug administration were included as TEAEs despite the low likelihood of being etripamil related; however, TEAE rates of hypotension or syncope did not materially change.

To summarize, during direct measurement of BP, the rate of symptomatic hypotension possibly related to etripamil across all 1,610 unique patients in phase 3 studies was 0.2%; and by TEAE collection, the rate of syncope possibly related to etripamil was 0.1%.

Hypotension is a potential adverse effect for IV adenosine and IV/oral beta blockers (eg, metoprolol and propranolol) and CCBs (eg, diltiazem, verapamil). For example, transient decreases in BP (systolic <90 mm Hg and/or diastolic <60 mm Hg) occurred in 5% to 10% of SVT patients treated with IV verapamil.^4^ Similarly, hypotension was the most commonly reported adverse event associated with IV diltiazem, with symptomatic hypotension occurring in 3.2% of patients.^5^ In 2 small studies (N = 15-33), symptomatic hypotension was observed after treating SVT patients with oral diltiazem and propranolol.^1^

The limitations of this analysis include the absence of BP measurements during the outpatient self-administration of etripamil during PSVT. Outpatient TEAEs could have been biased by self-reporting (TDs were directly medically supervised). Since BP data were collected only during supervised test dosing in sinus rhythm rather than during active PSVT episodes, the findings may not fully reflect the hemodynamic effects of etripamil in the intended clinical setting. However, as described previously, there were only 2 reported episodes of hypotension after etripamil use during PSVT that were unlikely caused by the medication and only a single episode of syncope with transient loss of consciousness after etripamil use during PSVT. Real-world evidence outside of clinical trial settings would be useful.

In contrast to IV and oral CCBs, intranasally administered etripamil demonstrates minimal BP reduction and only rare findings of hypotension or syncope in PSVT phase 3 studies. Together, these findings of negligible hypotensive effect support the safety of etripamil for patients to self-treat PSVT episodes, unsupervised, in the outpatient setting.

## Funding support and author disclosures

This study was funded by Milestone Pharmaceuticals Inc. Dr Ip has received compensation as steering committee member for Milestone Pharmaceuticals; and has received honoraria/speaking/consulting fees for Abbott Medical, Biotronik, Boston Scientific, and Medtronic Inc. Dr Stambler is a consultant for Milestone Pharmaceuticals. Dr Piccini has received personal fees from Milestone Pharmaceuticals outside of the submitted work. Drs Shardonofsky and Bharucha are employees and shareholders of Milestone Pharmaceuticals. Dr Camm has received compensation as a consultant for Milestone Pharmaceuticals; grants and personal fees from Acesion, Sanofi, Anthos and Johnson and Johnson; personal fees from Medtronic, Boston Scientific, Menarini, and Biotronik; and support from Anthos, Sanofi, Abbott, Glaxo SmithKline, and Johnson and Johnson. All other authors have reported that they have no relationships relevant to the contents of this paper to disclose.
